# The pros and cons of using automated sleep scoring in sleep research: Comparative analysis of automated sleep scoring in human and rodents: advantages and limitations

**DOI:** 10.1093/sleep/zsad275

**Published:** 2023-10-27

**Authors:** Abdelrahman Rayan, Anna B Szabo, Lisa Genzel

**Affiliations:** Donders Institute for Brain, Cognition and Behavior, Radboud University, Nijmegen, The Netherlands; Research Center on Animal Cognition (CRCA) and Brain and Cognition Research, Toulouse University, Toulouse, France; Donders Institute for Brain, Cognition and Behavior, Radboud University, Nijmegen, The Netherlands

**Keywords:** sleep, automatic sleep classification, machine learning

## Abstract

Sleep scoring plays a pivotal role both in sleep research and in clinical practice. Traditionally, this process has relied on manual scoring by human experts, but it is marred by time constraints, and inconsistencies between different scorers. Consequently, the quest for more efficient and reliable approaches has sparked a great interest in the realm of automatic sleep-scoring methods. In this article, we provide an exploration of the merits and drawbacks of automatic sleep scoring, alongside the pressing challenges and critical considerations that demand attention in this evolving field.

## Introduction

Sleep, which occupies approximately one-third of our lives, is a complex, and dynamic phenomenon characterized by continuous alterations between distinct states, each possessing unique properties, and distinct electrophysiological signature. These states can be visually identified by inspecting electroencephalogram (EEG), electromyogram (EMG), and electrooculogram (EOG) data. Notably, three major vigilance states can be distinguished: wake (desynchronized EEG activity patterns), non-rapid eye movement (non-REM) (comprising delta oscillations, spindles, and ripples), and rapid eye movement (REM) (wake-like brain activity associated with muscle atonia) [1]. However, it is crucial to recognize that within each of these states, further substates exist, such as quiet–active wakefulness, light/deep non-REM sleep, and phasic/tonic REM sleep [2, 3]. The identification of these states and their corresponding substates are critical for advancing our understanding of the functional aspects of sleep.

Remarkably, the majority of sleep stage scoring in both human and non-human animal sleep research continues to rely on manual assessment by expert scorers, a process that demands substantial labor and time, but also makes it difficult for newcomers to the field (Box 1). As a result, since the early years of sleep research in 1960s and 1970s, researchers have been striving to automate sleep scoring process (for a comprehensive overview of different methods used in automatic sleep scoring in rodents and humans, see Rayan et al. [4], Fiorillo et al. [5], and Phan et al. [6]). Despite the transition from hardware-based to software-based solutions and ultimately the utilization of various machine-learning algorithms, automatic sleep scoring has yielded limited contributions to our understanding of sleep architecture and there has been little progress observed in the accuracy of sleep scoring systems. This lack of progress is *particularly* pronounced in the clinical applications of sleep scoring systems, where automatic sleep scoring faces a markedly significant challenge [7]. Consequently, manual sleep scoring remains the prevalent method in these contexts.

Box 1: **A Newcomer’s Perspective on Sleep Scoring**

When joining the ever-expanding community of preclinical sleep researchers, one has to inevitably face the *Tower of Babel* of this field, as the absence of standardized rodent sleep scoring criteria means every researcher has their own *scoring language*.

This issue for a newbie first reveals itself when trying to differentiate between segments of the seemingly identical, dizzying lines on the monitor that the EEG signal looks like for a beginner. Of course, you eventually get to the point where a simple glance would suffice to tell sleep stages apart, but this requires training, which, in the absence of standardized criteria, is not unlike a round of whispering game. Indeed, scoring methods used in a laboratory are passed down from researchers to students and, at one point, from one student to another over the years—with the phrase “it is all slightly subjective” being thrown around every so often. In this process, the criteria go through gradual but substantial changes as each trainer will also transmit their attempts at ameliorating the analysis or complying with new trends in the field (e.g. how to mark the transition at the border of slow-wave sleep to REM sleep or how to score microarousals during sleep). On the one hand, it leads to potentially improved scoring strategies as decades of experience are being distilled over the years with the added layers of knowledge and observations from each participant in this peculiar whispering game. However, as an impressive number of hours are recorded even by a single rodent sleep study, digging through the data often requires long hours of meticulous sleep scoring from several members of a laboratory. Therefore, having varying scoring methods across team members eventually leads to decreased inter-rater coherence and potentially biased results.

While switching to automatic methods may improve the intra-team coherence issue, the ordeal has not finished. Once the analysis is over, you have your statistics in order, your figures painstakingly arranged and colored, and you get to the part where you would want to know how your results compare to previous publications. However, it soon becomes apparent that comparison is futile as the parameters used across research teams vary tremendously—be it epoch length, the number of slow-wave sleep stages, or once again the issue of transitional sleep. To speak of personal experience, this variability in scoring criteria is debilitating even at the level of highly specific (i.e. smaller) research topics only encompassing a handful dozen publications. At this point, you have two options: either let it go and publish—throwing yet another *scoring language* to the mix—or you brace yourself for redoing the whole analysis to comply with the criteria of at least one of the previous publications that you deem to be the gold-standard in your field. However, each previous methodology one stumbles upon has its own advantages, innovations but also its pitfalls—and there comes a point where it becomes all too tempting to try to create *the perfect method* by merging the positive components from the literature while ridding of the elements that seemed ambiguous. In the end, in spite of your efforts to ameliorate the situation, you end up aggravating it with another method stranger to both your own laboratory and to every other one, and that will eventually get lost in the turmoil around the ruins of the *Tower of Babel* that will remain until standardized sleep scoring criteria are introduced.

With the significant efforts involved in the development of automatic scoring methods and a lot of time already spent on these efforts without producing significant improvement in the output, it is crucial to thoroughly examine the potential advantages, disadvantages, and pitfalls associated with such endeavors. By examining these aspects, we can gain valuable insights into the implications and limitations of automatic scoring systems. Furthermore, the discussion addressing automatic sleep scoring also highlights the urgent need for a unified and standardized approach to sleep scoring in rodents, non-human primates, and other species, given their role in elucidating the neural underpinnings of sleep. Currently, a lack of consensus exists in this area, which hampers comparability and hinders progress of sleep research across different species, subsequently affecting the extrapolation of the results to clinical studies in humans. Addressing this gap by establishing a cohesive framework for sleep scoring methods would enable more reliable and robust comparisons facilitating a deeper understanding of sleep architecture across different species.

## Challenges in Standardizing Sleep Scoring

Human sleep states were initially established through the sleep scoring manual by Rechtschaffen et al. [1], followed by refinements provided in the sleep scoring manual of the American Association for Sleep Research [8]. The presence of well-defined and universally accepted criteria for different sleep states allows automatic scoring of human data to demonstrate a reasonable performance, particularly when applied to healthy participants (Box 2) [5, 6]. However, the successful implementation of such standard criteria and reliable automatic sleep scoring in humans requires the usage of large polysomnographic (PSG) setups. These set-ups incorporate measurements of brain activity, muscle tone, and eye movement and need to be applied by the expert, rendering them less suitable for home recordings, where simple wearable devices are preferred. The uncomfortable set of wires and the new environment (be it a laboratory or hospital) imposed on participants in sleep studies also suggest a potential lack of ecological validity to PSG studies, further deepening the issue [9].

Box 2: **Sleep States**

*REM sleep:* REM is a sleep state characterized by muscle atonia during sleep. In humans, it is associated with wake-like brain activity and vivid dreaming, while in rodents, it is characterized by the presence of predominant theta oscillation on the cortex and hippocampus.

*Phasic REM sleep:* Phasic REM sleep is a substate of REM sleep that is dominated by bursts of REMs.

*Tonic REM sleep:* Tonic REM sleep is another substate of REM sleep that is characterized by the absence of bursting eye movements.

*Non-REM (NREM) sleep:* NREM sleep is heterogenous sleep state comprised of several substates. It is generally characterized by the absence of REM and is further divided into different stages based on specific EEG patterns.

*Stage N1:* It is a transition state between wakefulness and sleep. In humans, it is identified by the disappearance of alpha oscillations in the EEG. This specific state has not been extensively examined I rodents, but an equivalent to N1 state in rodents could potentially be quiet wakefulness or NREM (for further details, see Rayan et al. [4]).

*Stage N2:* Stage N2 is slightly a deeper sleep state compared to N1. It is characterized by the presence of sleep spindles and K-complexes (global slow oscillations). Sleep spindles are commonly observed in humans and rodents during sleep.

*Stage N3:* This sleep stage is referred to as slow-wave sleep. It is characterized by the presence of high amplitude and low-frequency delta oscillations in the EEG. K-complexes and spindles also occur in this sleep state but can be hard to visualize due to the dominance of delta waves. All these oscillations are observed in humans and rodents.

In rodent sleep research, sleep is typically simplified to two main sleep states: REM and NREM (sometimes incorrectly referred to as slow-wave sleep). The classification of these states is primarily based on muscle tone (EMG) and the theta–delta ratio in the EEG or local field potential recordings. This simplified classification system overlooks the finer distinctions and substates observed during human sleep.

While human sleep stages have clearly been defined, and a consensus has been reached regarding the definition of the criteria used to identify various sleep states from the data obtained by PSG, there remains a lack of consensus regarding a standardized manual for sleep scoring in simpler set-ups with less recording parameters in humans, rodents, non-human primates, and other species. Indeed, the primary sleep architecture, comprising mainly of the two main states NREM and REM has been conserved on several branches of the evolutionary tree, including mammals, birds, cats, and, according to recent findings, potentially some cephalopods [10–15]. However, notable biological differences and variations exist in nomenclature across the different species. Particularly, the shorter sleep bouts and faster progression between the different sleep states [16] result in discrepancies in identifying the different sleep substates and their associated nomenclature (for the comprehensive overview of these differences and potential confounding factors in rodents’ sleep scoring, see [4, 17]). Addressing these variations and establishing an accepted framework for sleep scoring across different set-ups and species is imperative for achieving a comprehensive standardization in the sleep scoring field.

## Variations in Sleep Scoring Frameworks: Rodent Perspective

In most laboratories, rodent sleep is typically semiautomatically scored, with manual curations for any misclassifications or transitional sleep states. The process begins by setting a threshold value for the EMG activity to discriminate between wake and sleep periods. The sleep periods are then further classified into two primary sleep states non-REM and REM based on the theta–delta spectral power ratio and applying a corresponding threshold. Subsequently, an experienced sleep scorer visually examines the raw signal and reevaluates for any misclassifications, which frequently occur during the transition between the two sleep states. As a result, the majority of laboratories utilize a three-stage classification system (wake, non-REM, and REM), while employing a semiautomatic scoring system.

This process oversimplifies the complex and intricate sleep architecture observed in rodents, as documented by several early studies [3, 18, 19]. It reduces the multifaceted architecture to two primary sleep states, disregarding the presence of the transitional sleep state and other substates. For example, a transitional sleep state is characterized by the emergence of oscillations in the hippocampus and cortex in the theta/spindle range that persists for several seconds before transitioning into wake or REM sleep [3, 20]. Furthermore, relying solely on theta–delta ratio for sleep state scoring primarily aids in identifying the deep non-REM state, while it fails to accurately capture the light non-REM state. Moreover, the theta dynamics during REM sleep exhibit temporal variability, displaying bursting-like activity during phasic REM periods and differentiating it from tonic REM periods, which tends to be not considered [21].

Most automatic sleep scoring attempts have been conducted separately on humans and rodents, thereby failing to establish a correlation between sleep patterns in both species. Additionally, the absence of consensus regarding the definition of different sleep states and substates in rodents renders it challenging to extrapolate findings from rodent sleep studies to the human context and there have been misunderstandings considering, e.g. the use of slow-wave sleep in the past [17]. Finally, even when comparing rodent studies, significant differences arise from the divergences in methodological choices when constructing the sleep-scoring algorithm. Therefore, despite rodents serving as a crucial model for sleep research, our understanding of sleep in this species remains limited, with little effort devoted to enhancing our understanding of their sleep architecture and patterns.

Data availability and sharing among various laboratories pose a significant challenge in establishing a consensus for sleep scoring in rodents. While numerous repositories have been set up for human data e.g. OpenNeuro and National Sleep Research Resource, allowing for further exploration and validation by sleep and machine-learning researchers, similar initiatives are noticeably absent in rodent sleep research.

Several factors contribute to this gap. To start with, rodent sleep research attracts professionals from diverse backgrounds. These include machine-learning experts, dedicated rodent sleep researchers, and neuroscientists more focused on understanding physiological phenomena in the brain, such as memory consolidation during sleep, rather than on sleep itself. These researchers, coming from varied disciplines, often have distinct research questions and methodologies without a unified approach. For instance, a neuroscientist keen on deciphering the function of specific neurons during various sleep states might not focus on performing a comprehensive sleep classification. Instead, they may categorize sleep based on the electrophysiological signals relevant to their area of interest.

Furthermore, unlike humans, where sleep studies are primarily EEG-centric, rodent sleep research employs a variety of recording methods. Sleep researchers may prioritize EEG sleep signals, whereas neuroscientists might opt for deeper recordings from specific brain structures. This variability in data collection methods complicates the aggregation of sizable datasets. Coupled with the absence of well-defined sleep scoring criteria for rodents, establishing publicly available datasets becomes even more challenging.

In light of these considerations, we believe a critical step in compiling such a dataset is to reach a consensus on rodent sleep data scoring. Achieving this requires an open dialogue and collaboration among researchers from diverse disciplines.

## Advantages and Disadvantages of Automatic Sleep Scoring

In the following section, we will explore both advantages and disadvantages of automatic sleep scoring process (see Table 1).

**Table 1.** Strength and Weaknesses of Different Scoring Approaches

	Manual scoring	Automatic
Comparability of results across labs	−	+^1^
Time/work effort	−	+
Detecting short states	−	+/−
Atypical data	+	−
Variance of electrode placement	+	−
Artifact detection	+	+/−

^1^if same algorithm is used and same training dataset.

### Advantages

- *Time/labor saving:* The primary motivation behind the development of automatic sleep scoring is the significant time and expert labor required for manual scoring. While human scorers could theoretically be trained within a few days, scoring a full night sleep recording in humans typically takes approximately 2 hours, and similar length of recording in rodents takes around 0.5–2 hours if the substates e.g. light versus deep non-REM or phasic and tonic REM substates, are *not* considered during the scoring. Consequently, manual sleep scoring could consume several weeks of the project’s timeline. While automatic sleep scoring has the potential to reduce this time by several magnitudes of order, the actual reduction is usually less than anticipated. Currently, the most reliable systems tend to be semiautomatic, involving initial manual threshold-setting and extensive manual pre- and post-curation, including artifact removal and correction of misclassification, respectively. Even fully automated scoring systems require some manual interventions before or after scoring, leading companies offer such services for substantial fees per recording session.
- *Comparability of results:* Manual scorers may not always agree on the classification of every epoch, particularly when dealing with mixed or pathological sleep states. Even if the same scorer assesses the same recording session twice, slight variations can occur due to slight differences in attention to detail and concentration levels. By contrast, algorithm-based scoring generates consistent results as long as the sample size/training dataset is sufficiently large and the algorithm does not incorporate random walks. Therefore, employing automatic sleep scoring can significantly enhance the comparability of the results across different laboratories and projects.
- *Identification of substates:* The major sleep states can be further divided into substates, each serving potentially distinct functions and exhibiting unique electrophysiological properties. However, in case of rodents, these substates are often exceedingly brief, sometimes often lasting only a few seconds. Consequently, manual or current semiautomatic scoring systems typically overlook or fail to identify these substates. Thus, the classification and the potential discovery of new substates represent a significant advantage of the automatic scoring systems. Nevertheless, one drawback of machine-learning algorithms is that they are primarily designed to detect significant variations or unambiguous states, potentially overlooking subtle differences between substates unless explicitly trained to capture them.

### Disadvantages and caveats

- *Atypical data*: The main drawback of automatic scoring is its handling of atypical data. Although such data may not be as prevalent as normative sleep data, they can significantly impact the sleep-scoring process or the final diagnostic decision for human patients. This includes recordings obtained from patients, animal models of neurological disorders, or pharmacological interventions, among other factors that alter the fundamental sleep signatures or structure. For instance, an algorithm trained solely on standard sleep data might misinterpret seizures as artifacts, undermining the reliability of the automatic sleep scoring. While manual scoring might be more time-consuming when dealing with atypical data, the human experts; however, are more adept at adapting to anomalous events or conditions that occur during recording. They can readily adjust and prioritize the scoring criteria based on the unique features exhibited during the recording or after the intervention and identify other pathological conditions. This adaptability extends to identifying changes in the ongoing predominant oscillations such as spindles and delta oscillations, or a general alteration in the state’s properties or their sequence. As an example, modifications of serotonin levels, either through medications like selective serotonin reuptake inhibitors or genetic manipulations of rodents’ serotonin systems, can result in increased a state characterized by low muscle tone but wakeful brain activity, resembling quiet wakeful state [22, 23]. While current semiautomatic systems tend to classify such instances as Non-REM (focusing on muscle tone feature), human experts (focusing on brain activity) would score it as wake. One potential solution to this problem is to ensure that atypical data is included during the training phase of the network. Additionally, advancements in the field of artificial intelligence and artificial generalized intelligence, such as the utilization of language models and generative pretrained transformers or semantic-based algorithms (Box 3), hold promise for the development of more innovative solutions that exhibit enhanced human-like cognitive abilities, which could greatly improve the classification of atypical data.
- *Less flexibility:* Automatic sleep scoring methods demonstrate optimal performance when applied to data obtained by standard acquisition protocols. It is important to note that any changes in the electrode position can significantly impact the occurrence and the appearance of sleep state indicator oscillations e.g. delta oscillations in non-REM sleep, thereby affecting the classification accuracy of the automatic sleep scoring. In human sleep research, both Rechtschaffen et al. and AASM scoring criteria require specific electrode placements. Since human electrode placement is typically limited to the surface of the skull, complying with requirements is relatively straightforward. However, in the case of intracranial recordings in rodents or in humans, electrode placement is determined by the specific research question or pathological condition, leading to much greater variability in the electrode placement scheme. To facilitate the standardization of standard automatic sleep scoring in rodents, it becomes necessary to establish additional standard criteria including standard electrode placement locations. However, this may not always be feasible due to the small size of the rodent skull. Additionally, a consensus must be reached regarding the most advantageous electrode locations. Interestingly, locations that are currently less commonly used, such as olfactory bulbs in rodents, may prove to be more reliable for sleep scoring [24].
- *Lack of current standards:* To leverage automatic sleep scoring, it is essential to establish a unified system that is universally adopted or to ensure that different methods are validated against the same set of standards, enabling comparability of the results across studies. However, the current landscape lacks a consensus on scoring criteria specifically designed for rodents. Numerous attempts have been made to develop automatic sleep scoring. However, the lack of standard criteria makes it challenging to evaluate and compare their performance precisely. Consequently, without standardized framework for assessing the efficacy of these systems, the aforementioned advantage of automatic sleep scoring remains elusive in the current approaches.

Box 3: **Conceptual Future Directions**

*Deep ensemble learning:* Deep learning networks are renowned for their adaptability and scalability, attributes that enable them to adjust effectively to training data. However, their adaptability often stems from the use of stochastic training algorithms. As a result, they can be sensitive to the specific characteristics of the training dataset, rendering them susceptible to issues of bias and variance. Although contemporary advancements in deep learning aim to mitigate such concerns, the unique nature of each training session can yield divergent weight sets, consequently leading to variable predictions. This propensity for fluctuation, known as high variance, presents significant challenges when the objective is to craft an automatic sleep scoring method that predicts sleep states with high accuracy. One potential solution to this problem is ensemble learning, which involves the concurrent training of multiple models. By subsequently integrating their predictions using techniques such as bagging and boosting, ensemble learning not only mitigates the variance but also enhances overall prediction performance, outstripping what any single model could achieve. However, deep ensemble learning, despite its potential to refine the automatic sleep-scoring process, is not without drawbacks. Specifically, it is computationally demanding, given the need to train multiple models. Additionally, it introduces a further layer of computational complexity, which can sometimes obscure result interpretation. It is worth noting that the advantages of deep ensemble learning are most pronounced when supported by a diverse and comprehensive training dataset; absent such a dataset, the benefits might be marginal.

*Unsupervised Classification:* Unsupervised classification stands out as a promising avenue in future sleep scoring, offering a data-driven approach to classification. Rather than relying on pre-labeled data, this method allows algorithms to discern underlying variable relationships autonomously, with subsequent post-processing curation of the results. Granting such autonomy to unsupervised algorithms can potentially mitigate human biases in sleep state labeling, offering enhanced adaptability and the opportunity to uncover previously unidentified sleep states. A compelling example of this direction is the recent study by Katsageorgiou et al. [36], where they employed a deep neural network for sleep classification in mice, revealing numerous substates.

*Semantic segmentation:* In the realm of sleep scoring algorithms, a fundamental challenge is to semantically discern which epoch, characterized by specific features, matches a certain sleep state. Recent times have seen the advent of numerous techniques, with semantic segmentation—initially conceptualized for image processing and classification—gaining substantial traction. In semantic segmentation, pixels from an image are systematically categorized into distinct classes. Facilitating this categorization, the U-net architecture emerged as a prominent entity within the family of fully convolutional networks [37, 38]. Studies involving human participants, utilizing the U-net architecture, have unequivocally shown its superiority over conventional classification methods [26, 29]. The integration of such advanced techniques into automatic sleep scoring for rodents, particularly when combined with expansive and representative datasets, augments the prospects of elevating the precision and dependability of subsequent automatic scoring models. Additionally, there is a potential for large language models to offer new solutions by harnessing semantic search or reasoning to refine classifications process [39]. However, it is noteworthy that large language models come with their set of challenges, being computationally intensive, necessitating robust infrastructure, and producing significant CO2 emissions in comparison to other models.

**Improving Feature Selection**: Feature engineering, an indispensable component of machine learning and automatic sleep scoring, transforms raw sleep signals into insightful features encapsulating the complexity of sleep patterns. This process begins with feature extraction, converting raw sleep data into a comprehensive set of descriptive features. These can span a diverse spectrum, from spectral characteristics, and statistical metrics to time-domain attributes.

In rodent sleep research, data collection serves varied purposes and employs different recording methodologies. Consequently, it is vital to pick up features for data analysis that resonate with the specific attributes of the dataset. For instance, researchers often link a surge in sigma power (9–17 Hz) to the emergence of spindles. Yet, while spindle presence correlates with elevated sigma power, an uptick in this power does not inherently signify spindle presence. Spindles also exhibit distinct spatial and topographical structures, factors that merit consideration during their detection [40, 41]. This discernment is pivotal, especially when distinguishing the substates of NREM sleep stage.

This principle also holds for other oscillations, such as delta and theta, which have their unique spatial organization structures [32]. Opting for pertinent features from a dataset can amplify the efficacy of sleep-scoring algorithms. Through the application of feature selection strategies, like filter, wrapper, or embedded methods, one can pinpoint the most telling features that significantly bolster sleep state classification given the dataset at hand.

## Key Considerations in Automatic Sleep Scoring for Reliable Rodent Sleep Analysis

When developing a new automatic sleep scoring system, it is vital to consider and evaluate certain factors [4]. Notably, the duration of sleep cycles differs significantly between rodents and humans. For instance, while human sleep cycles last between 60 to 120 minutes, those of rodents span roughly 10 minutes [16]. Due to these shorter cycles, rodents might spend less time in each fine-grained sleep stage such as light NREM or phasic REM states. For instance, their phasic REM periods often last just a few seconds [21]. Given the brevity of these states, many researchers classify REM sleep in rodents as a uniform and homogenous state, overlooking its inherent complexity and variety. This can make manually classifying these short sleep phases difficult, leading to their frequent exclusion and omission during manual scoring. Consequently, when comparing automated scoring techniques in rodents to human-based evaluations, there’s a risk of inadvertently introducing the biases of human scorers into the model [25]. Overlooking these brief sleep segments, particularly during the training phase of a neural network, can skew results. Therefore, an automatic scoring system that might appear to be less effective could, in reality, offer greater accuracy than manual methods in this scenario.

Secondly, sleep is a multifaceted and dynamic phenomenon, marked by transient events or events obscured by other ongoing brain oscillations [4]. Relying exclusively on human scorers to evaluate the precision of a classification algorithm can inadvertently ignore sleep’s inherent complexity. Even within human sleep research, condensing sleep data into 30-second epochs to discern the prevailing sleep state might lead to overlooking briefer sleep substates. Hence, it is imperative to adopt automatic systems that go beyond the traditional benchmarks of sleep classification. Such systems should be capable of revealing previously unidentified states and subtle variables that might elude human scorers. Fiorillo et al. [26] adopted a comparable strategy, leveraging the U-sleep algorithm to show that with appropriate training, algorithms can operate effectively without being strictly bound to the conventional AASA guidelines.

Thirdly, the methods behind automatic scoring algorithms are often designed to identify and classify based on the dominant state within an epoch. This can inadvertently overlook the complexity of specific epochs, especially those characterized by mixed states. In contrast, human scorers, with their intuitive judgment, might be more adept at recognizing such heterogeneous elements within epochs. Consequently, the chosen epoch duration for classification ought to be tailored according to the distinct objectives of the classification. Adopting a blend of both long- and short-duration epochs in sleep classification could provide a trade-off between classifying the main sleep states and identifying the fine-grained sleep states [18, 19, 27]. Notably, the continuous evolution in the realms of machine learning and artificial intelligence is equipping us with innovative tools [28]. These tools e.g. U-sleep algorithm transcend the limitations of fixed time epochs, offering a more flexible and comprehensive approach to sleep analysis [25, 26, 29]

Fourthly, achieving accurate sleep scoring classification necessitates the careful identification and management of artifacts, as underscored in previous studies [3, 30]. These artifacts can originate from a myriad of sources, be it the recording apparatus or intrinsic sleep patterns like microarousal states [31]. Unfortunately, during sleep classification, microarousal states are often neglected and predominantly labeled as wake phases. Compounding this issue is the polyphasic sleep pattern in rodents, characterized by more frequent awakenings than in humans. This leads to a more prevalence of artifacts in rodents. Therefore, it is imperative to approach these artifacts with discernment, ensuring they are accurately differentiated and addressed during the classification process.

Finally, unlike in humans, rodent research often resorts to invasive implantation techniques or depth electrodes to examine the neural underpinnings of sleep and memory. The choice and placement of electrodes can significantly influence sleep scoring and the potential features viable for microstate classification. This becomes particularly pertinent when recordings are from a solitary brain region. It is noteworthy that the majority of sleep stages are classified predominantly in the low-frequency spectrum (<20 Hz), exhibiting variations across the brain’s anteroposterior axis [32]. Hence, even marginal shifts in electrode positions might impact sleep classification, more so in automated systems (as observed by Fang et al. [33]). For instance, studies that exclusively implant an electrode in the hippocampus, such as those by Winson [34] and Costa-Miserachs et al. [35], may not capture delta spectral dynamics in their entirety, given their prominence at frontal electrodes. Moreover, determining transitional sleep hinges on the consistent presence of spindles at the frontal electrodes, coupled with the onset of theta oscillations at posterior electrodes [3, 20]. Solely focusing on a single brain region for sleep classification risks obscuring the intricate dynamics of rodent sleep, which could lead to potential misclassification or imprecise state commencement identification during automatic sleep scoring.

## Conclusion

In conclusion, automatic sleep scoring holds great potential for enhancing efficiency, comparability, and identification of fine-grained sleep states. Despite the advantages offered by automatic sleep scoring, several challenges need to be addressed to fully harness its benefits. These challenges include handling atypical data, establishing standardized criteria and evaluation metrics, accounting for the unique characteristics of rodents’ sleep cycles, accommodating the heterogeneity within sleep epochs, considering the complexity of sleep, and effectively detecting and managing artifacts. Overcoming these challenges will require collaborative efforts between researchers across different domains, methodological advancements, and integration of newly developed artificial intelligence techniques (see Box 3). As the field progresses, incorporating advancements in artificial intelligence, such as using language models and generative pretrained transformers, may offer new avenues for more accurate and human-like experience capabilities during sleep scoring. By addressing these challenges and working towards standardization of sleep states’ definition, the potential of the automatic scoring system could be fully realized, providing researchers with powerful tools to unravel the mysteries of sleep and the neural mechanisms underlying memory consolidation.
